# Towards Computational Modeling of Ligand Binding to the ILPR G-Quadruplex

**DOI:** 10.3390/molecules28083447

**Published:** 2023-04-13

**Authors:** Xiaotong Zhang, John Barrow, Tanja van Mourik, Michael Bühl

**Affiliations:** 1 EaStCHEM School of Chemistry, University of St Andrews, North Haugh, St Andrews KY16 9ST, UK; 2 School of Medicine, Medical Sciences and Nutrition, Institute of Education in Healthcare and Medical Sciences, University of Aberdeen, Aberdeen AB25 2ZD, UK

**Keywords:** G-quadruplex, potential of mean force, ligand with high flexibility

## Abstract

Using a combination of unconstrained and constrained molecular dynamics simulations, we have evaluated the binding affinities between two porphyrin derivatives (TMPyP4 and TEGPy) and the G-quadruplex (G4) of a DNA fragment modeling the insulin-linked polymorphic region (ILPR). Refining a well-established potential of mean force (PMF) approach to selections of constraints based on root-mean-square fluctuations results in an excellent agreement between the calculated and observed absolute free binding energy of TMPyP4. The binding affinity of IPLR-G4 toward TEGPy is predicted to be higher than that toward TMPyP4 by 2.5 kcal/mol, which can be traced back to stabilization provided by the polyether side chains of TMPyP4 that can nestle into the grooves of the quadruplex and form hydrogen bonds through the ether oxygen atoms. Because our refined methodology can be applied to large ligands with high flexibility, the present research opens an avenue for further ligand design in this important area.

## 1. Introduction

**Figure 1:**
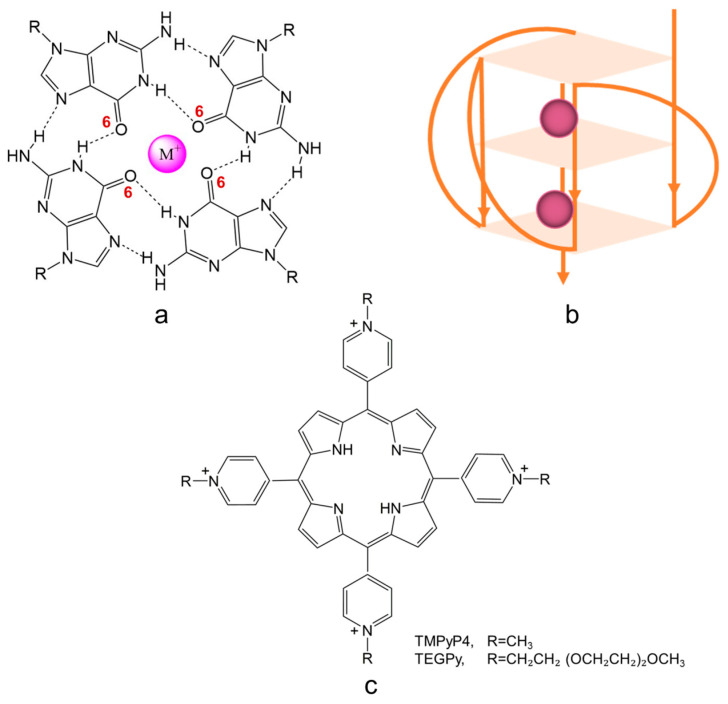
Schematic illustration of (**a**) a G-quartet stabilized by monovalent cations (such cations are usually coordinated to the inwardly pointing carbonyl oxygen atoms (O6) of the guanine bases forming the quartet); (**b**) a G4 structure with parallel conformation; and (**c**) G4 binding ligands with aromatic structural features; R=CH_3_ or CH_2_CH_2_ (OCH_2_CH_2_)_2_OCH_3_, representing TMPyP4 and TEGPy, respectively.

A G-quadruplex (G4) is a secondary structure of nucleic acids that are formed within specific repetitive guanine-rich DNA sequences. Rather than pairwise aggregation leading to the well-known double helix motif, four guanine moieties bind with each other, forming a G-quartet. Several G-quartets stack to form a G4 structure, which is stabilized by monovalent cations, located in the central cavity, either parallel with a quartet (coordinated to the carbonyl oxygens pointing inwards) or in between two successive quartets (see Figure 1a). Depending on the loop structure of the DNA strands involved, G4 DNA can adopt three conformations: parallel (see Figure 1b), anti-parallel, and hybrid [1,2,3].

Previous experiments have shown that G4 structures may be associated with gene regulation when they form in the promoter region, the DNA sequence to which proteins bind to initiate transcription [1,4,5,6,7,8,9,10]. Of particular interest is the formation of G4 structures in the insulin-linked polymorphic region (ILPR), as the G4 structure formed in ILPR (ILPR G4) may regulate the transcriptional activity of insulin [11,12,13,14,15,16,17]. Although the reason for the altered insulin gene expression by the ILPR G4 is unclear, previous studies suggest that the transcription factor Pur-1 can bind to G4 structures within the ILPR, thereby enhancing the recognition of RNA polymerase in the promoter region [11]. To further investigate the relationship between the ILPR G4 and gene regulation in situ, it is necessary to explore bespoke ligands, such as small molecules that display high affinity towards the ILPR G4 DNA. Because parallel conformations are more likely to prevail in the presence of intracellular molecular crowding [1,18,19], ligands with a high affinity for parallel G4s are particularly desirable.

The past decade has seen widespread interest in the development of potent G4 ligands. Among these ligands, TMPyP4 (5,10,15,20-tetra(N-methyl-4-pyridyl) porphyrin, Figure 1c), a tetra-cationic porphyrin derivative, has been well studied owing to its high affinity towards the external G-quartet of G4s [1,20,21,22,23]. Although it is controversial whether TMPyP4 displays selectivity towards different G4 structures, its aromatic structural characteristic and high sensitivity to the G-quartet make it a prominent candidate in ligand design. To improve the ability of ligands to differentiate between different G4 structures, side chains can be introduced into the design of ligands to further interact with G4s through groove and loop binding [23,24]. One such ligand that has been studied experimentally is TEGPy (tetra-[(ethylene glycol)-pyridyl]-porphyrin) (Figure 1c) [25,26]. Apart from the same polyaromatic core as TMPyP4, TEGPy comprises four PEG (polyethylene glycol) arms that may have additional interactions with the grooves or loops of the ILPR G4 structure, making it a promising ligand model for studying higher specificity binding to the ILPR G4 DNA.

Experimentally, ligand affinity can be estimated from the equilibrium binding constants obtained by several methods, including surface plasmon resonance (SPR) [27] and isothermal titration calorimetry (ITC) [28]. Nevertheless, it is challenging for experimental methods to determine the binding affinity with specific binding modes, especially when the difference in binding affinity between different binding modes is small. In addition, it is difficult to assess the dynamics of specific interactions between ligands and target molecules. Thus, computational approaches such as free-energy simulations are invaluable for providing quantitative mechanistic information on binding specificity and affinity. They can also predict binding constants for as-yet-unknown ligands, which could build the basis for the in silico design of inhibitors or regulators.

Recently, Deng et al., reported the binding affinity of c-MYC G4-quindoline in two binding pockets using the potential of mean force (PMF) approach [29]. Their results are in general agreement with those obtained by SPR, which demonstrates the validity of the PMF approach to study the c-MYC G4-quindoline system. Previous computational studies have demonstrated the superiority of free-energy simulations in characterizing the thermodynamics of ligand-G4 binding [29,30,31]. However, most of the studies have focused on relatively small ligand systems, and there is little research on ligands such as TEGPy that are large, highly flexible, and display multiple binding modes simultaneously towards G4s. This is because the flexibility and large size of such ligands will increase the conformational sampling spaces, which require extensive computational resources and may result in convergence problems for the free-energy simulations.

In this study, we investigate the absolute binding free-energy and ligand interactions of ILPR G4-ligand systems using two porphyrin derivatives through the Root-Mean-Square Fluctuation (RMSF)-based PMF protocol. By evaluating the RMSF of the ligands, we make a rational selection of restraint atoms to assist the system in reaching equilibrium in free-energy perturbation. This makes the free-energy approach applicable to large, flexible G4-ligand systems, which otherwise pose big challenges to this type of free-energy simulation. With our refinement of the PMF protocol, we can accurately calculate the absolute binding free energy of the ILPR G4-TMPyP4 system, and rationally predict the absolute binding free energy of the large and flexible TEGPy ligand towards the ILPR G4 structure. Interactions between the four side arms of TEGPy and the groove of the ILPR G4 structure will also be highlighted. The comparison between TEGPy and TMPyP4 allows assessment of the ligand affinity with different binding modes towards the ILPR G4 DNA, which is essential for probe design and for assisting experiments to unravel the relationship between G4 formation and regulation of the human insulin gene.

## 2. Results and Discussions

**Conformational sampling of the ILPR G4-ligand systems using unconstrained MD simulations.** To fully understand the structural features and dynamic behavior of the ILPR G4-ligand systems, unconstrained MD simulations were performed. The structural fluctuations of the ILPR G4-TMPyP4 and the ILPR G4-TEGPy systems during the simulations are analyzed using the RMSD and RMSF values, with the reference structure being the equilibrium structure obtained from the unconstrained MD simulations.

The geometry of the ILPR G4 and the ILPR G4-TMPyP4 complex displayed slight conformational changes during the 5 ns unconstrained MD simulation (Figure S1a). Both systems reached equilibrium at ~500 ps and maintained their structures throughout the simulation with a structural fluctuation of RMSD ~4.5 Å, suggesting that the π-π interactions between the G4 and TMPyP4 moieties are well conserved. The computed RMSF of the G4 (Figure S1c) varies from loop regions (larger fluctuation) to quartets (smaller fluctuation), indicating a relatively rigid conformation in the quartet. This is due to the π-π interactions between the outer quartet and TMPyP4, which in turn lead to similar small magnitudes of fluctuations in the atoms close to the aromatic ring of TMPyP4 (Figure S1d). Due to the additional side arms, the conformation of TEGPy is more flexible than that of TMPyP4, which in turn affects the G4 structure. As shown in Figure S2a, both the ILPR G4 and the ILPR G4-TEGPy systems present a structural fluctuation, with an RMSD ~5 Å, which is slightly larger than that of the ILPR G4-TMPyP4 system. However, the RMSF shows that TEGPy undergoes unequal structural changes compared to TMPyP4 (S2d vs. S1d). This is apparent in the π plane, where the structural fluctuation of each atom is supposed to be very similar to each other. This finding highlights the importance of selecting the correct restraint atoms in the constrained MD simulations, or more specifically, the free-energy perturbation in restraining highly flexible ligands, such as TEGPy.

**Figure 2:**
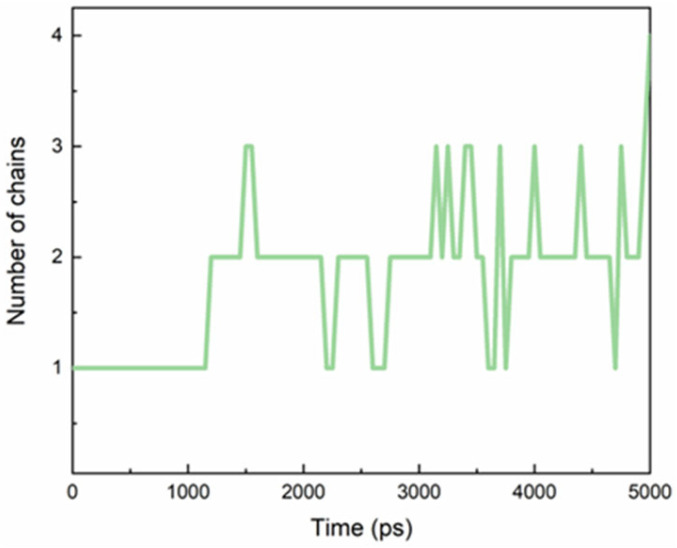
The evolution of the average number of TEGPy arms that nestled in grooves of the ILPR G4.

TEGPy was designed as a ligand for G4 structures because the four side arms are expected to interact with the G4 grooves [26]. We started our unconstrained MD simulation from a structure where these four arms had been placed manually into the four grooves. The average number of side chains that nestled into the grooves during the MD simulation was evaluated by computing the distance between the outermost ether oxygen atom of the ligand and its nearest nitrogen atom from the guanine base of the quartet (Figure 2), assuming an arm is tightly bound when the arm-groove distance is below 3.5 Å. During the MD simulation, however, it turned out that only two or three of the arms are tightly bound in their grooves; the others are more flexible. This finding suggests the possibility that ligands with fewer side arms can be expected to bind just as well as, or even better than, ligands with four side arms. This conjecture will need to be confirmed by subsequent studies. For a detailed discussion of H-bonding between the side arms and the grooves, read the next paragraph.

**Absolute binding free energies for the ILPR G4-ligand systems from the RMSF-derived PMF approach.** The absolute binding of free energies $\Delta G_{bind}^{{}^{\circ}}$ of the ILPR G4-TMPyP4 and ILPR G4-TEGPy systems and the individual components of these are listed in Table 1. The computed absolute binding free energy of the TMPyP4-ILPR G4 system is −9.18 (±0.48) kcal⋅mol^−1^, which is in good agreement with the previous ITC results (−9.2 kcal⋅mol^−1^) [32]. This validates the RMSF-based PMF approach, which accurately predicts the binding affinity between the ILPR G4 and its ligands. The computed absolute binding free energy of the TEGPy-ILPR G4 system is −11.50 (±0.59) kcal⋅mol^−1^, approximately 2.3 kcal⋅mol^−1^ larger (in absolute terms) than that of the complex with TMPyP4. This increase in the absolute binding free energy corresponds to a ca. 50-fold increase in binding affinity at room temperature (the computed free binding energies correspond to affinities of 0.005 nM^−1^ and 0.240 nM^−1^ for TMPyP4 and TEGPy, respectively). Since TEGPy might exhibit multiple binding modes towards the ILPR G4, the enhanced binding strength compared to TMPyP4 may be attributed to the additional binding, i.e., loop or groove binding. According to a recent study involving fluorescence correlation spectroscopy [33], TMPyP4 is indicated to bind slightly stronger to the 5′ ends of human telomeric DNA sequences than to the 3′ ends used in the present study (by a factor of ca. 7, albeit with quite a large uncertainty). Our predicted increase in binding affinity on going from TMPyP4 to TEGPy may thus be somewhat overestimated but should still be noticeable.

The contributions of each component of the absolute binding free energy in both systems were examined and are listed in Table 1. The computed free energy changes for restraining the ligand in its bound/unbound state are very close to the two different ligands (−5.62 kcal⋅mol^−1^ and −5.73 kcal⋅mol^−1^ for the TEGPy and TMPyP4 complex in its bound state; 9.54 and 9.43 kcal⋅mol^−1^ for the TEGPy and TMPyP4 complex in its unbound state). Clearly, the strength in restraining the ligand to the bound state, or the unbound state (solvent) is very similar, and the main contribution to the difference in the ligand affinity of the two ligand systems comes from the potential of mean force.

**Figure 3:**
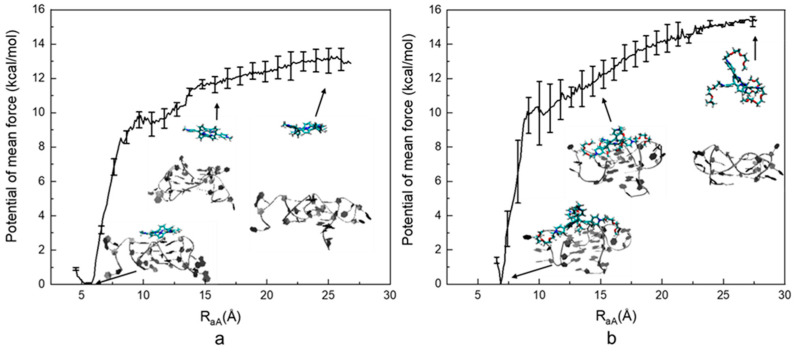
Computed potential of mean force of the ILPR G4-ligand systems. (**a**) PMF profile of the ILPR G4-TMPyP4 system. (**b**) PMF profile of the ILPR G4-TEGPy system. Intermediate conformations of the DNA-ligand complex at different reaction coordinates are also shown, where the DNA molecule is represented by new ribbons, and TMPyP4 is shown by licorice representations.

As indicated in Figure 3, the computed PMF of the ILPR G4-TMPyP4 system is −12.88 ± 0.43 kcal⋅mol^−1^. The lowest energy occurs at ~5.5 Å, which is a rather deep minimum, with the free energy rising steeply on either side. As R_aA_ increases, the energy goes up rapidly until R_aA_ reaches ~8 Å. After that, the energy rises at a moderate rate and then levels off after ~20 Å, indicating the ligand is dissociated from the ILPR G4. The computed PMF of the TEGPy system is −15.42 ± 0.53 kcal⋅mol^−1^, which is 2.54 kcal⋅mol^−1^ higher than that of the TMPyP4 system. The lowest energy occurs at ~6.5 Å. As R_aA_ increases to ~10 Å, the computed PMF of the TEGPy system rises more rapidly than that of the TMPyP4 system, as shown by the steeper slope in the profile, indicating a higher free-energy change. Then, as R_aA_ increases to 12.5 Å, the PMF gradually increases, suggesting weaker interactions between target and ligand molecules compared to the π-π interactions observed at the previous stage. This phenomenon suggests that weaker interactions, such as hydrogen bonding, occur in the TEGPy system, leading to a higher affinity of the ligand. The energy profile finally levels off after 25 Å, where TEGPy is fully dissociated from the ILPR G4 DNA and reaches the solvent. It can be seen from the first intermediate conformation of the TEGPy system (R_aA_~6.5 Å) that the ligand displays multiple interactions (groove and loop binding as well as π-π interactions with the ILPR G4), which can explain the higher ligand affinity. In addition, compared to TMPyP4, TEGPy requires a longer distance to dissociate from its target, as some of the side arms can still interact with the external quartet or flanking bases of the outermost loop region. As exemplified in the third intermediate conformation in the TEGPy complex (R_aA_~20 Å), a side chain of TEGPy is still close to the external quartet of ILPR G4, whereas TMPyP4 has already approached the solvation environment at a similar distance. It is clear that the loop and groove interactions formed in the ILPR G4-TEGPy system enable a higher affinity of TEGPy towards the ILPR G4 DNA.

**The higher ligand affinity of TEGPy is attributed to the loop and groove binding.** To further understand the loop and groove interactions formed in the ILPR G4-TEGPy system, the number of intermolecular hydrogen bonds along the ligand dissociation was evaluated in Figure 2. An interaction was considered to be an intermolecular hydrogen bond if the distance between a potential donor and an acceptor atom is below 3.5 Å, and the donor-hydrogen-acceptor angle is larger than 150°. The number of intermolecular hydrogen bonds formed in the TEGPy system was measured using the H-bond plugin in VMD.

**Figure 4:**
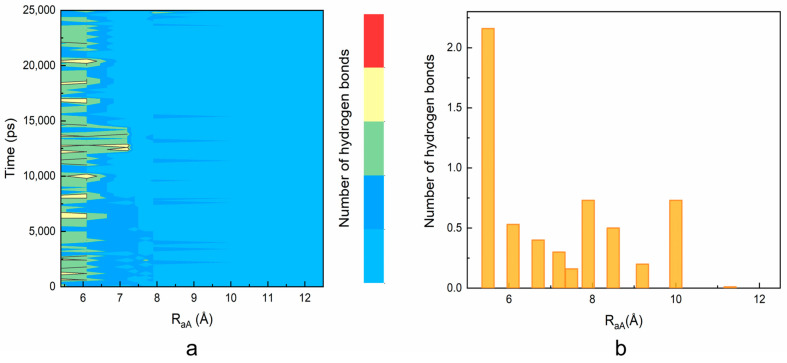
The evolution of the number of intermolecular hydrogen bonds in the TEGPy-ILPR G4 system along the ligand dissociation. (**a**) The number of intermolecular hydrogen bonds as a function of time and R_aA_. The number of hydrogen bonds (ranging from 0 to 4) is indicated using a color scale. (**b**) The average number of intermolecular hydrogen bonds against R_aA_. The number of hydrogen bonds is computed at each R_aA_ value as an average over the 25 ns constrained MD simulations.

As shown in Figure 4a, two or three intermolecular hydrogen bonds are maintained between the ILPR G4 and TEGPy during the 25 ns constrained MD simulation with a R_aA_ ~5.5 Å, which is in general agreement with that of the unconstrained MD simulation (Figure S3). This result validates the choice of restraint atoms, as the overall number of hydrogen bonds is not affected by the external restraint force. When R_aA_ elongates to 7 Å, the number of hydrogen bonds decreases to one and mostly ranges between zero and one when R_aA_ extends to 8 Å.

**Figure 5:**
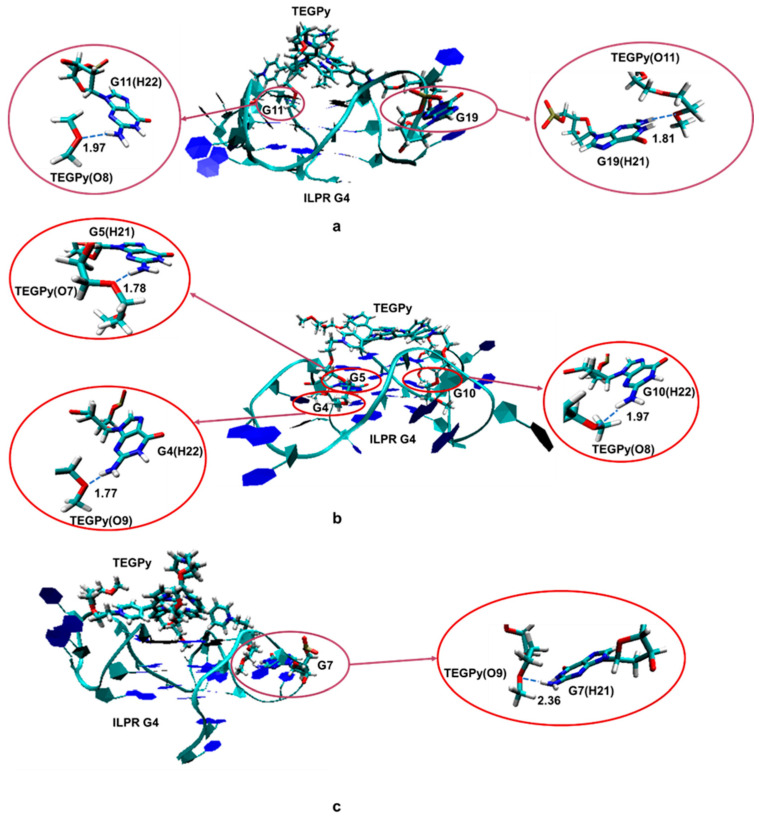
Schematic illustration of hydrogen bonds formed in the ILPR G4-TEGPy complex (see text for description of individual interactions (**a**–**c**). The guanine bases participating in the hydrogen bonding with TEGPy and TEGPy are represented as licorice, whereas the remaining part of ILPR G4 DNA is shown in new ribbon. All distances presented in this figure are labeled with dashed lines and given in Å. See Figure S5 for the numbering scheme of the O atoms.

Some hydrogen bonding between the ILPR and TEGPy is maintained at 10 Å, which confirms that TEGPy needs to reach a long distance from the ILPR G4 to fully dissociate from it. The average number of hydrogen bonds as a function of R_aA_ is shown in Figure 4b. In general, the number of hydrogen bonds decreases as the distance increases. There are two slight rebounds at around R_aA_ = 8 Å and 10 Å and the number of hydrogen bonds levels off after 10 Å. We also investigated which regions of the ILPR G4-TEGPy system are involved in hydrogen bonding (Figure S4). Overall, the hydrogen bonding between TEGPy and the ILPR G4 is maintained within R_aA_~10 Å, indicating the important role of hydrogen bonding in the ILPR G4-TEGPy binding. Some characteristic snapshots illustrating hydrogen bonding in the ILPR G4-TEGPy system are shown in Figure 5. There are two hydrogen bonds shown in Figure 5a. One occurs between the hydrogen atom (H22) of the amino group in guanine (G11, first quartet layer) and the oxygen atom (O8, Figure S5) of the ether group in TEGPy with a distance of 1.97 Å. The other one is formed between hydrogen atom H21 of G19 (flanking guanine base in the loop) and the oxygen atom (O11) of the outermost ether group of the ligand (1.81 Å). In Figure 5b, hydrogen bonds exist between H21 of G5 (first layer guanine base), H22 of G4 (middle layer guanine base), H22 of G10 (middle layer guanine base), and O7, O9, and O8 of TEGPy, respectively. It can be seen from the given snapshots that O8 (oxygen atom of the outermost ether group in one arm of TEGPy) is involved in hydrogen bonding with two quartet layers in the groove region of the quadruplex. Similarly, O9, located in another side chain of TEGPy, interacts with both the loop region (G7) and groove region (G4) of the quadruplex through hydrogen bonding. In Figure 5c, there is a single hydrogen bond formed between the hydrogen atom (H21) of G7 (flanking guanine base in the loop) and the oxygen atom (O9) of the ligand (2.35 Å). The oxygen atoms participating in the hydrogen bonding are shown in Figure S5. These snapshots from the constrained MD simulation, together with the previous results, illustrate that groove binding and loop binding exist simultaneously in the ILPR G4-TEGPy system through hydrogen bonds, which contribute to the higher ligand affinity of TEGPy towards the G-quadruplex compared to TMPyP4.

How could the design of ILPR ligands be useful in therapeutic applications? One promising treatment for diabetes is the transplantation of tissue containing the insulin-producing beta-cells from donor pancreases into the liver of the diabetic patient [34]. Because there is a drastic shortage of donor pancreas material, it is imperative to find an alternative source of beta-cells that can be transplanted. To date, nobody has managed to produce a replenishable source of beta-cells that are suitable as a therapy for diabetes, although there have been successful protocols established that can create beta-cells in vitro [35,36]. Stem cells could offer an ideal solution to this problem. One approach that could enhance stem cell differentiation is the use of novel molecules, such as ILPR ligands, which can alter the expression of important genes such as insulin. With the ILPR sequence being unique in the human genome, it is a promising target for creating a ligand that could affect insulin gene expression and potentially offer a pathway to novel therapeutics and diabetes therapies. The uniqueness of the ILPR sequence also offers the opportunity for small molecule delivery of therapeutics that will only target the beta-cells and no other cells in the body. Any small molecule that is able to bind to the ILPR DNA sequence would be an enabling technology for the following applications: (1) the delivery of anti-apoptosis drugs to the beta-cells: the breakdown (apoptosis) of the beta-cells is a major causative factor in diabetes, so stopping this process early could offer a preventative treatment; (2) the delivery of beta-cell stimulating factors: therapeutic agents could be delivered to the beta-cells to stimulate them to multiply—this could alleviate diabetes symptoms in patients who still have a small number of beta-cells remaining in their pancreas; and (3) imaging and identification of beta-cells: whilst not strictly therapeutic, the non-invasive ability to identify and image the beta-cells in patients would be a significant aid in the clinical management of diabetes.

## 3. Materials and Methods

Due to the lack of a crystal structure of the ILPR G4, a related DNA fragment with a parallel conformation (Protein Data Bank entry 3CDM) was modified to provide the initial DNA model. Using the monomeric conformation of 3CDM, we mutated its sequence from [d(TAGGGTTA(GGG)_3_)_2_] to [d(TAGGGTGTGGG)_2_] (the monomeric sequence of the ILPR that was found to be most likely to form G4s and is associated with the high transcriptional activity of insulin) [37,38], using Chimera [39]. The experimentally refined 3CDM structure is not of the best quality but should be sufficient for our molecular modeling study (which involves structure minimizations and MD simulations). Both ligands were bound to the 3′ ends of the ILPR G4 (the natural choice for TEGPy because the loop ends at the 5′ ends are expected to interfere with the binding of the TEGPy side chains to the DNA).

We used nucleic acids parameters from ff94 [40] as implemented in Gromacs [41,42] for the ILPR-G4 ligand systems in solution. There have been some debates about the best force field to use for DNA [43,44,45,46]; the force field we are using has been validated for G4 systems in current research [47,48,49], as well as in the present work (see ESI and Figure S6 for further validation). GAFF2 [50] was used to model the ligands. The partial charges of the ligands were obtained by the AM1-BCC charge model [51]. The starting structure of the ILPR G4-ligand systems in the unconstrained MD simulation was fully optimized in the gas phase at the GFN2-xTB level of semiempirical density functional theory [52]. Each ILPR G4-ligand complex was initially placed in the center of a cubic periodic box and saturated with TIP4P water molecules. The distance between the outermost solute atoms and the nearest face of each box was set to 10 Å. The box size for the ILPR G4-TMPyP4 and the ILPR G4-TEGPy systems is 63 Å and 70 Å, respectively. Considering the negatively charged phosphate backbone of DNA, 16 sodium ions were added to the solvent to neutralize the system. The particle mesh Ewald (PME) method was used, with a cut-off of 10 Å, to describe the long-range electrostatic interactions of each system. Initially, 5000 energy minimization steps using the steepest-descent algorithm were conducted, followed by 100 ps pre-equilibration in the NVT ensemble followed by 100 ps in the NPT ensemble, respectively. Subsequently, a 5 ns production run was performed on each system at 300 K.

**Figure 6:**
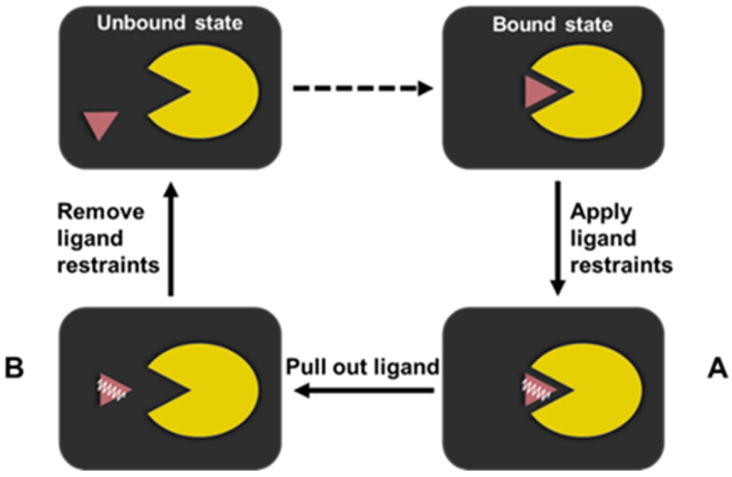
The RMSF-based PMF protocol based on the thermodynamic cycle adopted by the ILPR G4-ligand system. The ILPR G4 and its binding ligand are represented by the yellow Pac-man and purple triangle, respectively. The harmonic restraints applied to the ligand are indicated by a white spring.

An RMSF-based PMF protocol employing the thermodynamic cycle of the ligand dissociation was used to compute the absolute free binding energy of the ILPR G4-ligand systems (Figure 6). This is based on the protocol originally proposed by Karplus and co-workers [53] and adapted by Deng et al. [29]. The bound state of the ligand systems is one equilibrated structure from the previously unconstrained MD simulation.

**Figure 7:**
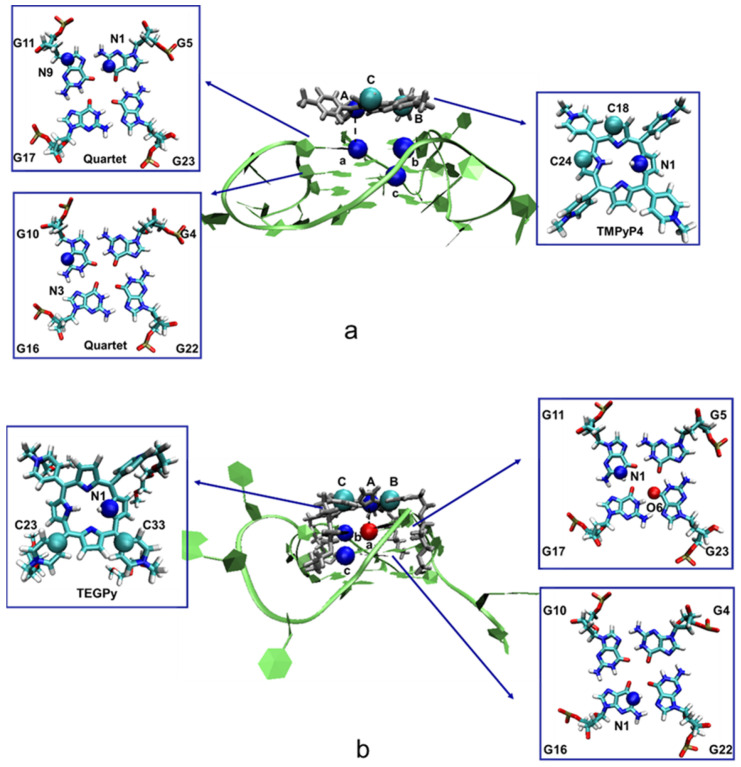
Schematic illustration of the restraint atoms in the ILPR G4-ligand systems. (**a**) Restraint atoms in the ILPR G4-TMPyP4 complex. Atoms A (N1), B (C24) and C (C18) are located close to the aromatic core of TMPyP4, whereas a (N1, G5), b (N9, G11), and c (N3, G10) are in the first and second layer of the ILPR G4 structure. (**b**) Restraint atoms in the ILPR G4-TEGPy complex. Atoms a, b, and c and A, B, and C are located at the aromatic core of TEGPy and the quartets of the ILPR G4 structure, respectively. G stands for the guanine base. Note that restraining atoms with unfavorable RMSFs can lead to insufficient restraint of the ligand orientation, resulting in convergence problems in the constrained MD simulations (see Figure S9 and Table S1).

Starting from the bound state, the orientation and position restraints of the binding ligand were gradually switched on (state A) via a series of *λ* values ranging from 0 to 1 (0 represents no restraints, whereas 1 denotes full restraints). The restraint atoms are selected by the evaluation of the all-heavy-atoms RMSF with respect to the bound state structure. Atoms that are expected to be in a more rigid region, but have larger computed RMSF, are considered good candidates for restraining the ligand orientation (Figures S1 and S2), because using these atoms larger fluctuations of the ligand (which would need long simulation times for correct sampling) can be suppressed. The appropriate choice of restraint atoms is key for applying the chosen protocol to this type of ligand (Figure 7). For the TMPyP4-ILPR G4 system, 16 *λ* values were used in addition to 0 (0.01, 0.015, 0.05, 0.075, 0.10, 0.15, 0.20, 0.25, 0.35, 0.45, 0.50, 0.60, 0.70, 0.80, 0.90, and 1.00). These values are selected with reference to Deng’s work, where 14 *λ* values were used for quindoline (a ligand with a similar size to TMPyP4) [29]. As TEGPy is larger and more flexible than TMPyP4, more *λ* values were needed. Thus, for the ILPR G4-TEGPy system, 25 *λ* values are used in addition to 0 (0.01, 0.02, 0.03, 0.04, 0.05, 0.06, 0.07, 0.08, 0.09, 0.10, 0.12, 0.14, 0.16, 0.20, 0.25, 0.30, 0.35, 0.40, 0.45, 0.50, 0.60, 0.70, 0.80, 0.90, and 1.00). For each *λ* window, 5 ns equilibration at 300 K was performed, followed by a 5 ns production run. The difference in free energy upon switching on the restraints from the bound state to state A can then be estimated using the Bennett Acceptance Ratio (BAR), a procedure implemented in Gromacs [54], which also furnishes an estimated error based on the fluctuation of ∂H/∂λ (for $\Delta G_{restr}^{bound}$ in Equation (1)).

From state A to B, umbrella sampling along the reaction coordinate R_aA_ (Figure 7) was applied in both systems, with the ligand restraints kept at their full strength. For both ligand systems, there are 20 individual sampling windows, in which different values along R_aA_ were set. The initial conformation of each R_aA_ was generated by gradually pulling the ligand away from its binding target using the PULL code [55]. Conformations were saved every 1 Å. At each window, different values of R_aA_ were restrained with a force constant of 1000 kJ mol^−1^⋅nm^−2^ for an initial 5 ns equilibration phase, to allow the simulations to adjust to the restraints. Subsequently, a 20 ns production run was conducted at 300 K. An illustrative example showing the convergence of the mean force is shown in Figure S8 in the ESI. The PMF of the ligand-ILPR G4 systems was analyzed using the Weighted Histogram Analysis Method (WHAM) [56] as included in Gromacs. The statistical fluctuation of the free-energy change (ω(r) in Equation (1)) was evaluated twice for each ligand, namely by the standard deviation of repeating two 10 ns trajectories and by that of repeating four 5 ns trajectories. The largest of the resulting standard deviations was used as the final error estimate (see Figure S8 for details).

When the binding ligand escaped from the ILPR G4 and reached the bulk region (state B), all the restraints were switched off. The ligands can freely rotate in the solvent, and therefore, the free-energy change of the restraints in the solution can be estimated by the final term in Equation (1) (from state B to the unbound state). The absolute binding free energy of the ILPR G4-TMPyP4 and ILPR G4-TEGPy systems is computed by [29]

(1) $$\Delta G_{bind}^{{}^{\circ}}=-\Delta G_{restr}^{bound}-w\left(r^{*}\right)-k_{B}T\ln\frac{\int_{bound}e^{-w\left(r\right)k_{B}T}dr}{{\left(\frac{2\pi k_{B}T}{k_{r}}\right)}^{\frac{1}{2}}}+\Delta G_{restr}^{bulk}$$

(2) $$\Delta G_{restr}^{bound}=k_{B}T\ln\frac{\langle f{\left(H_{A}\left(p,q\right)-H_{B}\left(p,q\right)+C\right)\rangle }_{B}}{\langle f{\left(H_{B}\left(p,q\right)-H_{B}\left(p,q\right)-C\right)\rangle }_{A}}+C$$

(3) $$\Delta G_{restr}^{bulk}=-k_{B}T\ln\frac{C^{0}r^{*2}sin\theta_{0}sin\Theta_{0}{\left(2\pi k_{B}T\right)}^{3}}{8\pi^{2}{\left(k_{r}k_{\theta }k_{\phi }k_{\Theta }k_{\Phi }k_{\Psi }\right)}^{\frac{1}{2}}}$$

where the free energy of restraining the ligand at the bound state is expressed as $-\Delta G_{restr}^{bound}$. This can be estimated using BAR as shown in Equation (2), where f is the Fermi function $f\left(x\right)=1/(1+\exp\left(x/k_{B}T\right))$, and $C=k_{B}T\ln\frac{Q_{A\;}n_{B}}{Q_{B\;}n_{A}}$, $n_{A}$ and $n_{B}$ are the numbers of configurations generated in the states A (the bound state without restraints) and B (the bound state with restraints), respectively. p and q represent the coordinates and momenta of the Hamiltonian. The potential of the mean force of the dissociation of the ligand is determined by $w\left(r\right)$, where *r* is the current location of the ligand and r* is the location where the ligand reaches the bulk. This is to some extent ambiguous as long as the ligand is fully dissociated from its G4 target. For the ILPR G4-TMPyP4 and ILPR G4-TEGPy systems, r* is taken to be 20 and 22.5 Å, respectively (the position where the PMF has levelled off, see Figure 3).$\;k_{r}$ is the force constant of the harmonic restraint with a value of 1000 kJ⋅mol^−1^⋅nm^−2^. The free-energy change of restraining the ligand in the solvent is represented by $\Delta G_{restr}^{bulk}$, where $k_{\theta },k_{\phi },k_{\Theta },k_{\Phi }$ and $k_{\Psi }$ are the force constants for restraining the ligand orientation and position in both ILPR G4-ligand systems. $k_{\theta }$ = $k_{\phi }$ = $k_{\Theta }$ = $k_{\Phi }$= $k_{\Psi }$= 1000 kJ⋅rad^−1^⋅mol^−1^. These restraint values are the average values obtained from the previous unconstrained MD simulations. The uncertainty of the third term in Equation (1) (starting with −*k_B_T*) was estimated from the values of the integral at the upper and lower bounds of *r*, according to the procedure by Deng et al. [29].

## 4. Conclusions

We studied the absolute binding free energy and ligand interactions of the ILPR G4-ligand systems using a PMF-based protocol. Previous computational studies have demonstrated the usefulness of free-energy simulations in characterizing the thermodynamics of ligand-G4 binding [29,30,31]. However, most of the studies have focused on relatively small ligand systems and there has been limited research into ligands such as TEGPy that are large, highly flexible, and display multiple binding modes simultaneously towards G4s. This is because the flexibility and large size of such ligands increase the conformational sampling spaces, require extensive computational resources, and may result in convergence problems in the free-energy simulations. In this study, we found that TEGPy displays a rigid core and flexible side chains. A poor choice of restraint atoms in restraining such ligands can lead to improper free-energy perturbations. To overcome this issue, we have improved an RMSF-based PMF protocol to assist in selecting restraint atoms that confine the unnecessary fluctuations of atoms in the rigid region caused by the flexible part of the ligand, while maintaining the flexibility of the ligand. This novel protocol was validated for the ILPR G4 system by the excellent agreement of the computed absolute free binding energy of TMPyP4 (−9.18 ± 0.48 kcal⋅mol^−1^) with that obtained by the ITC experiment (−9.2 kcal⋅mol^−1^). Using this approach, we rationally predict the absolute free binding energy (−11.50 ± 0.59 kcal⋅mol^−1^) and binding modes of the flexible and large modular ligand, TEGPy, in the ILPR G4 system. The results show that TEGPy displays a higher ligand affinity towards the ILPR G4 (ca. 50-fold increase in the binding constant at room temperature), due to its side chains interacting with the loop and groove regions through hydrogen bonding. However, in contrast to the binding mode proposed previously, we found that only some of the four side arms fit into the groove regions simultaneously. This finding suggests that ligands with fewer side arms might be expected to bind just as well as, or even better than, ligands with four side arms. This possibility provides a new perspective for designing ILPR G4 ligands and may assist in interpreting the important role that G4 formation plays in the regulation of the insulin gene.

Classical simulations are routinely used to study ligand-receptor binding, usually involving fairly small ligands (typically ca. <100 atoms, as opposed to the 174 atoms of TEGPy) Application of these standard tools to large, highly charged and, in the case of TEGPy, highly flexible ligands, poses a substantial challenge. The successful demonstration that a more refined methodology from the literature can be applied to such ligands and G4 structures is a significant development, opening the avenue for further ligand design in this important field. The present research has helped to expand the limits on the complexity of receptors and ligands, further enhancing the applicability and usefulness of biomolecular simulations.

## Figures and Tables

**Table 1:** Absolute binding free energy in the ILPR G4- TMPyP4 and ILPR G4-TEGPy systems.

ILPR G4-Ligand Systems	$-\omega \left(r^{*}\right)$ (kcal·mol^−1^)	$-k_{B}T\ln\frac{\int_{bound}e^{-\omega \left(r\right)/k_{B}T}dr}{{\left(\frac{2\pi k_{B}T}{k_{r}}\right)}^{1/2}}$(kcal·mol^−1^)	$\Delta G_{restr}^{bulk}$ (kcal·mol^−1^)	$-\Delta G_{restr}^{bound}$ (kcal·mol^−1^)	$\Delta G_{bind}^{{}^{\circ}}$ (kcal·mol^−1^)
ILPR G4-TEGPy ^a^	−15.42 (±0.53)	−0.01 (±0.0006)	9.54 (±0.0)	−5.62 (±0.27)	−11.50 (±0.59)
ILPR G4-TMPyP4 ^a^	−12.88 (±0.43)	−0.02(±0.0007)	9.43 (±0.0)	−5.73 (±0.05)	−9.18 (±0.43)

^a^ The error bars are obtained from standard deviations between results from two repeating 10 ns trajectories in the umbrella sampling windows (see computational details).

## Data Availability

The research data supporting this publication can be accessed at https://doi.org/10.17630/9505b210-dfec-401c-b32d-9151ae3810e3 (accessed on 1 April 2023).

## Supplementary Materials

The following supporting information can be downloaded at https://www.mdpi.com/article/10.3390/molecules28083447/s1 (additional graphical and tabular material: Additional simulation details are shown in Figures S1 and S2; Additional simulation results are shown in Figures S3–S5; Figure S6: Force fields comparison between ff94 and parmbsc1; Figure S7: Convergence of mean constrained force; Error estimation for *ω*(r*) over repeating four trajectories are shown in Figures S8 and S9; Table S1: Computed free energy difference in restraining the ligand at the bound state of the ILPR G4-TEGPy system); pubXiaotong-G4-SI-*.pdb (files with coordinates of the full systems with ligands bound).
