# Supplementary material for: Towards Computational Modeling of Ligand Binding to the ILPR G-Quadruplex
Source: Molecules. 2023 Apr 13;28(8):3447. doi: 10.3390/molecules28083447 (PMC10145587; doi:10.3390/molecules28083447)
Supplement: Supplementary file 1 [file molecules-28-03447-s001.zip › molecules-2271806-supplementary.pdf]

Supplementary Material

Towards Computational Modeling of Ligand Binding to the ILPR G-Quadruplex

Xiaotong Zhang [1], John Barrow [2], Tanja van Mourik [1,*] and Michael Bühl [1,*]

[1] EaStCHEM School of Chemistry, University of St Andrews, North Haugh, St Andrews KY16 9ST, UK
[2] School of Medicine, Medical Sciences and Nutrition, Institute of Education in Healthcare and Medical Sciences, University of Aberdeen, Aberdeen AB25 2ZD, UK
* Correspondence: tanja.vanmourik@st-andrews.ac.uk (T.v.M.); buehl@st-andrews.ac.uk (M.B.)

Additional simulation details

Root Mean Square Fluctuation (RMSF)-based protocol

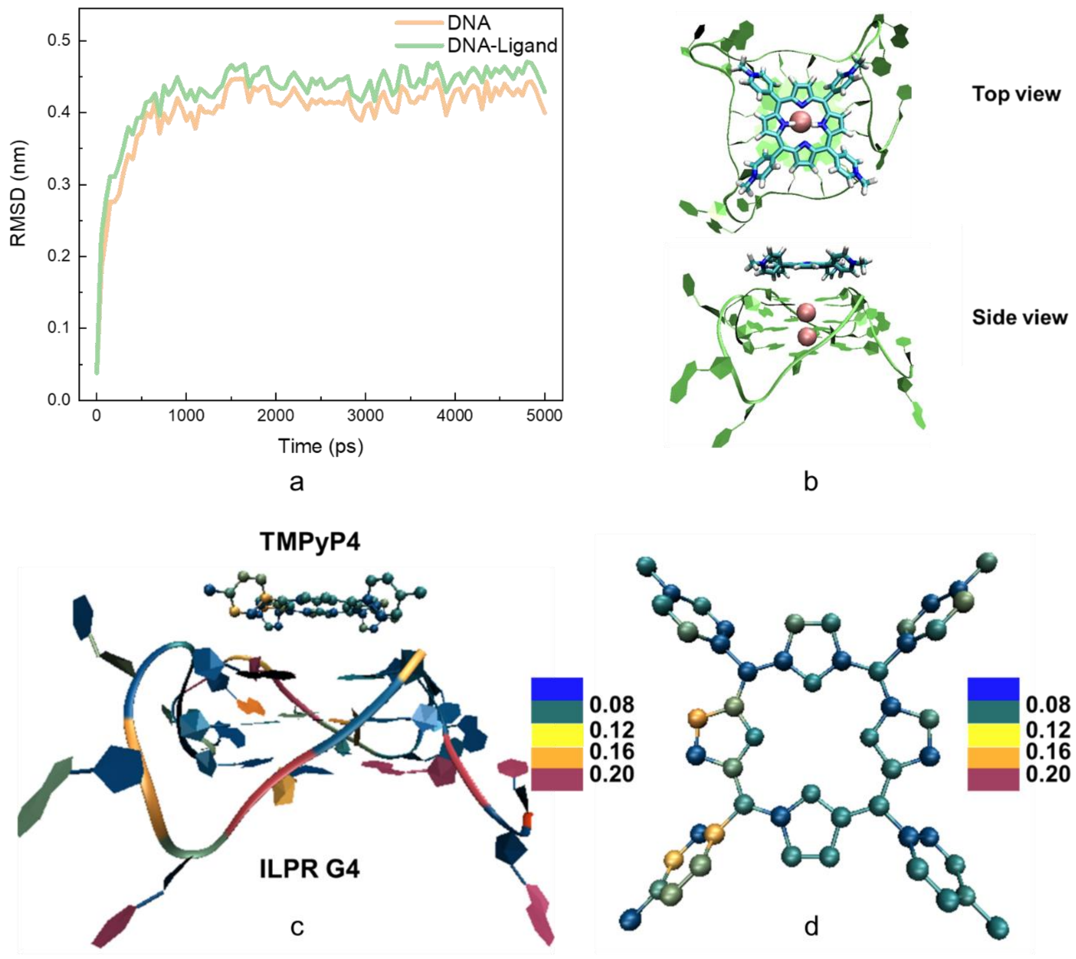

Figure S1. Results of the unconstrained MD simulation of the ILPR G4-TMPyP4 system. a) Computed Root Mean Square Deviation (RMSD) over all atoms (including hydrogens) of the ILPR G4 (DNA, orange line) and the ILPR G4-TMPyP4 (DNA-Ligand, green line) systems against time, where the reference structure is the initial structure used for MD simulations. b) The equilibrium conformation of the ILPR G4-TMPyP4 complex. The ligand, G4 and potassium ions are represented as liquorice, new ribbons, and spheres, respectively. c) Calculated Root Mean Square Fluctuation (RMSF) of all heavy atoms of the ILPR G4-TMPyP4 system relative to the initial structure for the MD simulation. All values are in nm and illustrated using colour scales. d) Computed RMSF of TMPyP4 with the reference structure being the optimised structure obtained by GFN2-xTB calculations.

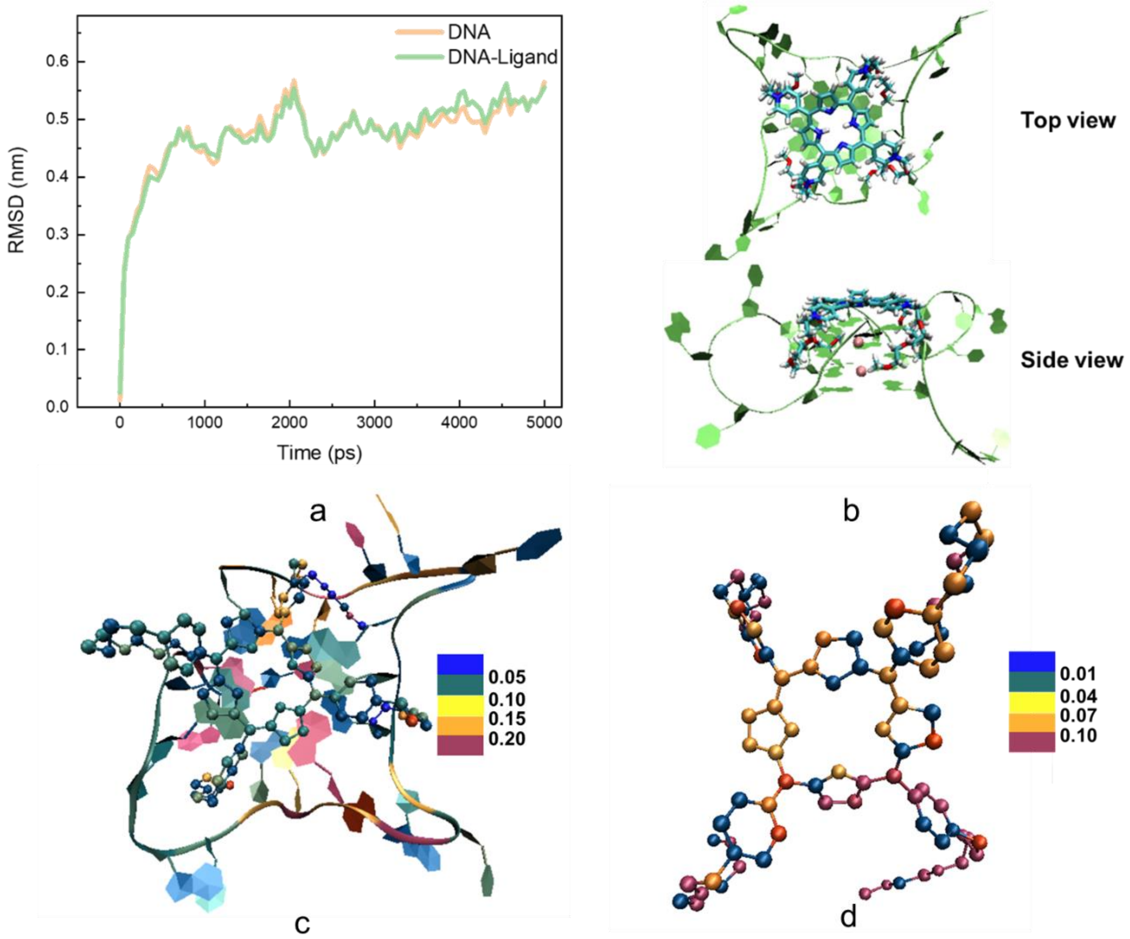

Figure S2. Results of the unconstrained MD simulation of the ILPR G4-TEGPy system. a) Computed RMSD of the ILPR G4 (DNA, orange line) and the ILPR G4-TEGPy (DNA-Ligand, green line) systems against time, where the reference structure is obtained by geometry optimization using GFN2-xTB. b) Fully optimised ILPR G4-TEGPy complex at the GFN2-xTB level, where the G4 DNA, TEGPy and potassium ions are represented as new ribbon, liquorice and spheres, respectively. c) Calculated RMSF of all heavy atoms of the ILPR G4-TEGPy system relative to the initial structure for the MD simulation. All values are in nm and illustrated using colour scales. d) Computed RMSF of the TEGPy with the reference structure being the optimised structure obtained by GFN2-xTB calculations.

Additional simulation results

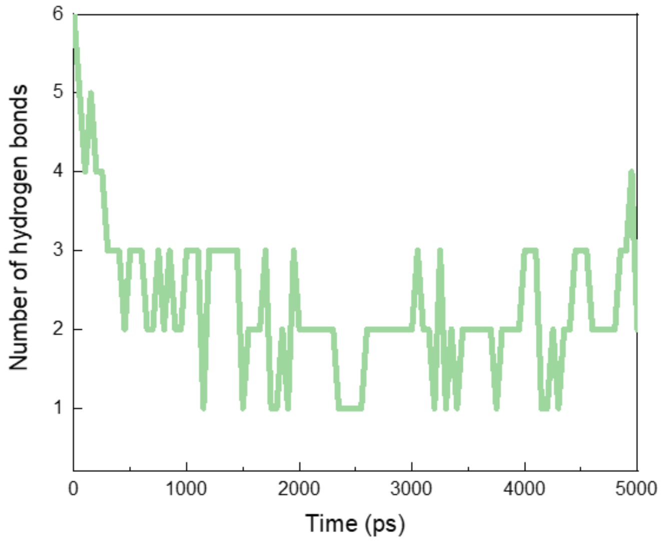

Figure S3. Number of intermolecular hydrogen bonds formed between the side chains of TEGPy and the grooves of the ILPR G4 DNA during the unconstrained 5 ns MD simulations.

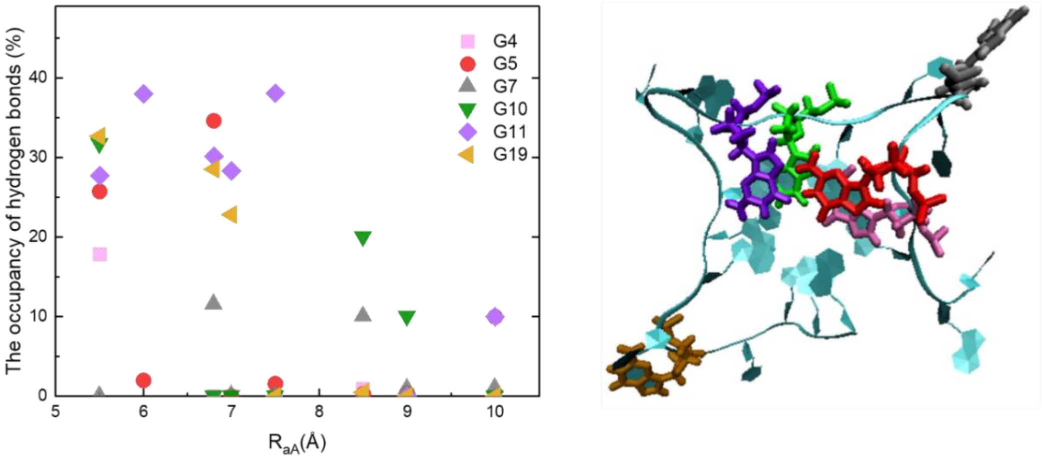

Figure S4. Schematic diagram of the evolution of the occupancy of hydrogen bonds in the ILPR G4 complex with R_{aA}. The guanine bases involved in hydrogen bonding are marked by index numbers and are represented by different colours and shapes. The figure shown on the right is the top view of the ILPR G4 structure, where the bases involved in the hydrogen bonding are represented as liquorice, and the rest of the quadruplex is shown in new ribbon.

As illustrated in Figure S4, six guanine bases participate in hydrogen bonding with TEGPy; two of these (G7 and G19) are flanking bases in loop regions, whereas the other four (G4, G5, G10, and G11) are in different quartet layers. Compared to the other four bases, G11 (located in the first quartet layer opposite to the starting strand) and G19 (the flanking base in the loop region near the end of the strand) are involved in the largest proportion of hydrogen bonding interactions with TEGPy.

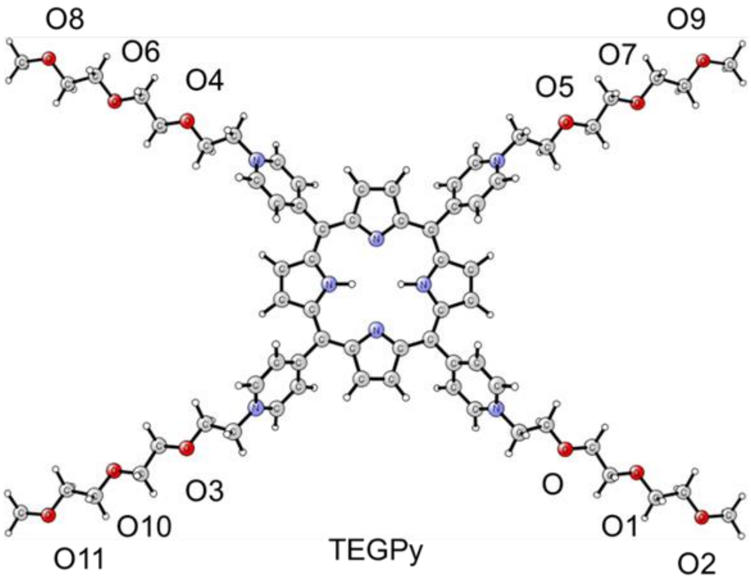

Figure S5. Schematic illustration of the oxygen atoms that act as hydrogen bond acceptors in TEGPy.

Force fields comparison between ff94 and parmbsc1

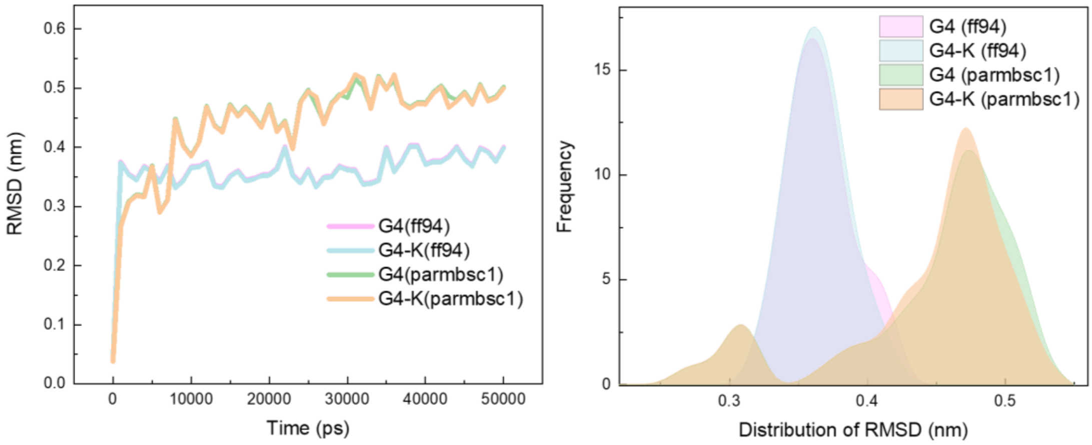

Figure S6. RMSD of the ILPR G4 system with ff94 and parmbsc1. RMSD of the heavy atoms of the ILPR G4, and the G4-K^+ complex with parameters from ff94 and parmbsc1(left). Distribution of the RMSD values with ff94 and parmbsc1. G4 and G4-K^+ stand for the heavy atoms of the ILPR G4 and the ILPR G4-potassium systems, respectively (right).

The performance of ff94 and parmbsc1 for the ILPR G4 system was compared through 50 ns unconstrained MD simulations. The structural fluctuations of G4 and G4-K^+ (two potassium ions that are initially coordinated in the central channel of the G4) were examined by calculating the RMSD of all heavy atoms (Figure S6). The reference structure for calculating the RMSD is the mutated crystal structure of 3CDM. According to Figure S6, both the conformation of the ILPR G4 and the complex of G4-K^+ displayed less fluctuation (roughly 1 Å) with the parameter set of ff94 compared to parmbsc1. This can also be deduced from the RMSD distributions, where the central value of ff94 and parmbsc1 are located at around 0.35 and 0.45 nm, respectively. Both results suggest the superiority of ff94 for evaluating the dynamics of the ILPR G4 system.

Convergence of mean constrained force

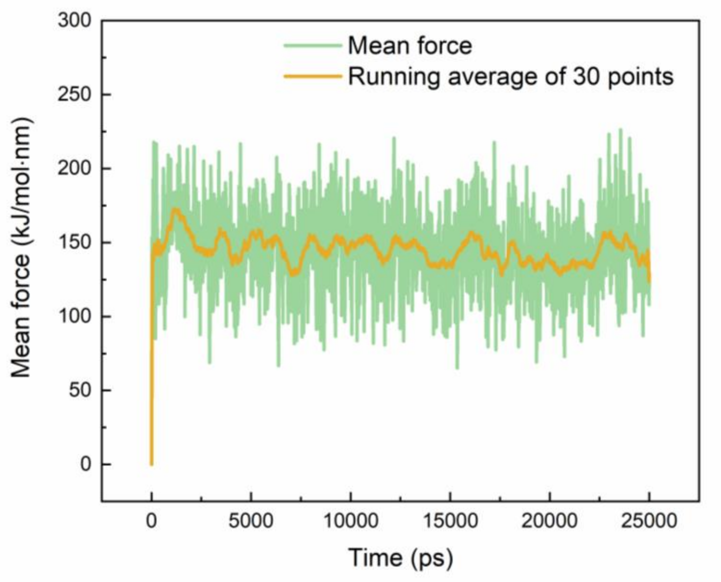

Figure S7. The evolution of the mean force along 25 ns constrained MD simulation when the force between TEGPy and ILPR G4 was strongest (R_{aA} = 10 Å). The running average of the mean force was computed every 30 points.

The convergence of the mean constrained forces has been monitored through their running averages, ensuring that there is no drift and that the final cumulative (overall) averages are stable. This is illustrated for one example in Figure S8, where the running average appears to be converging after ca. 3 ns.

Error estimation for ω(r*) over repeating four trajectories

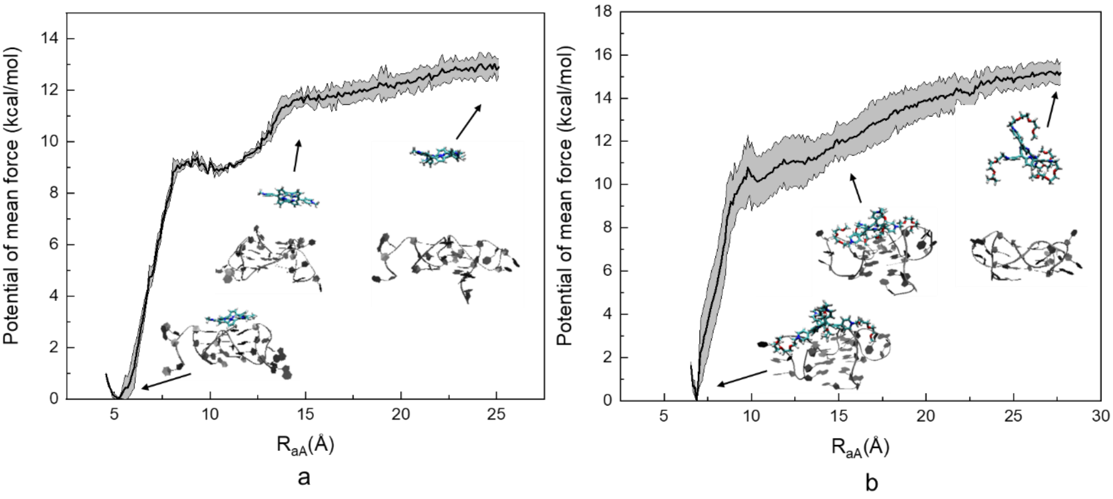

Figure S8. Computed potential of mean force of the ILPR G4-ligand systems using four trajectories of 5 ns. a) PMF profile of the ILPR G4-TMPyP4 system. b) PMF profile of the ILPR G4-TEGPy system. Intermediate conformations of the DNA-ligand complex at different reaction coordinates are also shown, where the DNA molecule is represented by new ribbons, and TMPyP4 is shown by liquorice representations.

When the PMFs are computed over two trajectories of 10 ns each, $\omega(r^*)$ values of 15.42±0.12 kcal/mol and 12.88±0.43 kcal/mol are computed for TEGPy and TMPyP4, respectively. For the analysis of 4 trajectories of 5 ns each, the corresponding values are 15.16±0.53 kcal/mol and 12.89±0.32 kcal/mol, respectively. For the final values we used the

means from the first analysis, and the largest of the two errors for each ligand, affording the values given in Table 1 in the main paper.

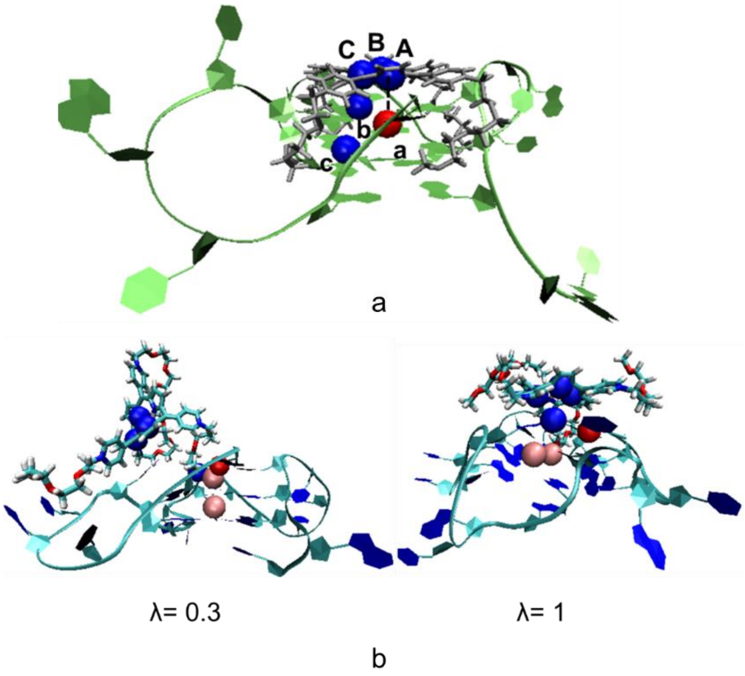

Figure S9. Structures of the ILPR G4-TEGPy complex with random restraint atoms. a) The restraint atoms of the LPR G4-TEGPy complex. The ligand and target are represented by silver liquorice and lime ribbons, respectively. Atoms A, B and C are located close to the aromatic core of TEGPy, whereas atoms a, b, and c are located in the different layers of the ILPR G4. b) Structures of the ILPR G4-TEGPy system with different restraint λ values using random restraint atoms. $\lambda = 1$ indicates the ligand is fully restraint. The two potassium ions are represented by pink spheres.

Table S1. Computed free energy difference in restraining the ligand at the bound state of the ILPR G4-TEGPy system ($-\Delta G_{restr}^{bound}$).

Restraint components at the bound state	Restraint energy (kcal·mol^{-1})
Position restraints	7.32 (±0.15)
Orientation restraints	29.54 (±0.73)
$-\Delta G_{restr}^{bound}$	−34.94 (± 0.60)

Structures of G4-ligand complexes

The initial geometry and final snapshots from our unconstrained MD simulations of the G4-TMPyP4 and G4-TEGPy bound states are provided separately in pdb format to facilitate setup of other ligands with this system.
